# Modeling Combined Chemotherapy and Particle Therapy for Locally Advanced Pancreatic Cancer

**DOI:** 10.3389/fonc.2015.00145

**Published:** 2015-07-06

**Authors:** Marco Durante, Francesco Tommasino, Shigeru Yamada

**Affiliations:** ^1^Department of Biophysics, GSI Helmholtzzentrum für Schwerionenforschung, Darmstadt, Germany; ^2^Department of Physics, Trento Institute for Fundamental Physics and Applications (TIFPA), National Institute for Nuclear Physics (INFN), University of Trento, Trento, Italy; ^3^Research Center Hospital for Charged Particle Therapy, National Institute of Radiological Sciences (NIRS), Chiba, Japan

**Keywords:** pancreatic cancer, protontherapy, heavy ion therapy, chemoradiotherapy, gemcitabine

## Abstract

Pancreatic ductal adenocarcinoma is the only cancer for which deaths are predicted to increase in 2014 and beyond. Combined radiochemotherapy protocols using gemcitabine and hypofractionated X-rays are ongoing in several clinical trials. Recent results indicate that charged particle therapy substantially increases local control of resectable and unresectable pancreas cancer, as predicted from previous radiobiology studies considering the high tumor hypoxia. Combination with chemotherapy improves the overall survival (OS). We compared published data on X-ray and charged particle clinical results with or without adjuvant chemotherapy calculating the biological effective dose. We show that chemoradiotherapy with protons or carbon ions results in 1 year OS significantly higher than those obtained with other treatment schedules. Further hypofractionation using charged particles may result in improved local control and survival. A comparative clinical trial using the standard X-ray scheme vs. the best current standard with carbon ions is crucial and may open new opportunities for this deadly disease.

## Introduction

Pancreatic cancer (PC), usually ductal adenocarcinoma, is the fourth cause of cancer-related death in USA (1) and the only cancer for which deaths are predicted to increase in Europe for both men and women in 2015 (2). Even after surgery, the mortality from PC is very high. Radiotherapy is used for radical treatment in locally advanced unresectable tumors (LAUPC), generally in combination with chemotherapy, or prior to surgery for potentially resectable malignancies. However, prognosis remains very poor, with <5% of patients surviving for 5 years after diagnosis (3). This makes PC a priority for finding better ways to control it and better treatments. Early tumors usually do not cause symptoms, so that the disease is typically not diagnosed until it has spread beyond the pancreas itself, either with distal metastasis or with infiltration in the neuroplexus. This is one of the reasons for the poor survival rate. Moreover, PC is very hypoxic (4), which makes it radioresistant and promotes epithelial–mesenchymal transition; is resistant to apoptosis; and presents a dense tumor stroma, which acts as a barrier against immune cells, preventing immune suppression (5).

Radiobiology studies suggest that charged particle therapy (CPT) using protons or carbon ions is more effective for treatment of PC than X-rays. In fact, accelerated ions have a reduced oxygen enhancement ratio (OER), and are therefore exquisitely effective against hypoxic tumors (6). Moreover, high doses of densely ionizing radiation elicit a strong immune response, which can be exploited to destroy not only the primary tumor but also distal metastasis (7). Carbon ion radiotherapy (CIRT) is currently performed in only two centers in Europe (HIT in Germany and CNAO in Italy) and none in USA (where many centers use protons only for CPT), but much more experience has been accumulated in Asia, especially at the National Institute for Radiological Sciences (NIRS) in Chiba, Japan. A recent external review of 20 years of CIRT at NIRS highlighted treatment of PC as the most promising application of CIRT, with results clearly superior to any other treatment modalities, especially for LAUPC (8).

Based on these very promising preliminary Japanese results, the US National Cancer Institute (NCI), in his efforts to promote CIRT in USA, issued a solicitation for a prospective randomized phase-III trial comparing CIRT to X-ray therapy for LAUPC in combination with chemotherapy, having survival as main endpoint^1^. This trial may provide the first evidence of a superiority of CIRT in a common and deadly cancer. Planning of the trial is complicated by the many different variables – not only radiation quality but also chemotherapy regime, fractionation, and treatment plan. Here, we review all the current results in treatment of LAUPC and use a mathematical model to describe the dependence on survival on the biological effective dose (BED) with X-rays and CPT in combination with chemotherapy.

## Materials and Methods

### Data collection

**Figure 1:**
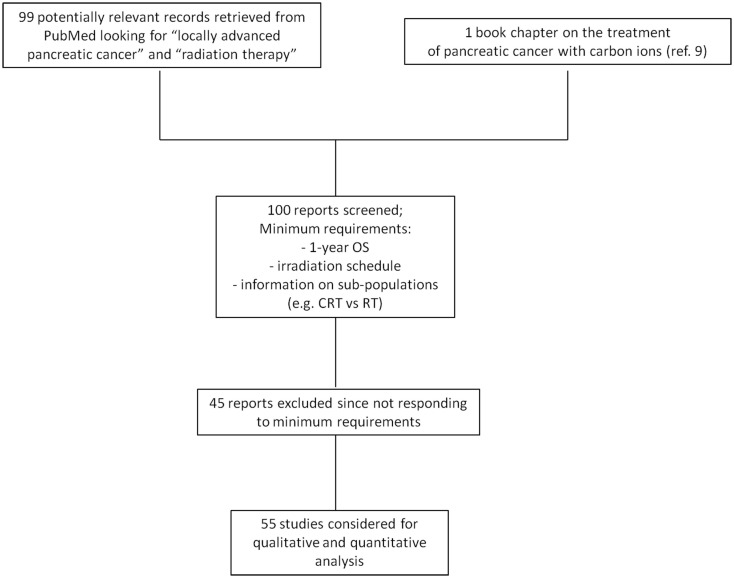
**Diagram summarizing the selection criteria of the studies included in the analysis**.

We searched the literature for all data available on radiotherapy, chemotherapy, and combined treatments. The research criteria and outcomes are summarized in the diagram shown in Figure 1. The patient populations generally consist of adults with adenocarcinoma histology, locally advanced tumor presentation, and generally tumors not in direct contact to duodenum and stomach. Radiotherapy included conformal radiotherapy (3DCRT), intensity-modulated radiation therapy (IMRT), stereotactic body radiotherapy (SBRT), protontherapy, and CIRT. Data from CIRT are limited to the NIRS experience and include data as yet only published in the institute annual report and in a recent book (9). Adjuvant, neo-adjuvant, or concomitant chemotherapies were all included in the search, using different drugs. Our data collection was compared with a recent meta-analysis of radiochemotherapy in LAUPC (10), and has been updated on April 2015.

### Modeling

To compare the largely variable fractionation and chemotherapy schedules reported in the literature, we used the common quantity of BED (11), which has been extended to chemotherapy to quantify the effect of the drug in terms of radiation-equivalent dose (12). Because many published papers have short follow-up, and not all endpoints are reported, we concentrated on the 1-year overall survival (OS). We assumed that the overall 1-year survival probability OS is a combination of the survival probability following the radiation (RS) and chemotherapy (CS) treatment, i.e.,

(1) $$\text{OS}=\text{CS}+\text{RS}\left(\text{1}-\text{CS}\right)$$

Equation 1 implies a purely additive effect of chemotherapy and radiotherapy in the treatment of LAUPC. The dose–response for the OS probability can be expressed with the same functions used for the tumor control probability: Poisson, logistic, or probit models (13). We elected to use the logistic function, which is based on the linear-quadratic model, following the recent model of chemoradiation treatment in bladder cancer (14). Thus, we wrote:

(2) $$\text{RS}=\frac{1}{1+\exp\left[4\gamma_{\text{50}}\left(1-\frac{\text{BED}}{D_{\text{50}}}\right)\right]}$$

where γ_50_ is the normalized dose-response gradient and *D*_50_ the BED corresponding to a survival in a radiotherapy only treatment of 50% at 1 year.

Combining Eqs 1 and 2, we finally obtain

(3) $$\text{OS}=\frac{1+\text{CS}\cdot \exp\left[4\gamma_{\text{50}}\left(1-\frac{\text{BED}}{D_{\text{50}}}\right)\right]}{1+\exp\left[4\gamma_{\text{50}}\left(1-\frac{\text{BED}}{D_{\text{50}}}\right)\right]}$$

In a recent analysis of chemoradiation therapy in LAUPC, Moraru et al. (15) used a radiosensitization factor in the BED formula and fitted the LAUPC 1 year OS data with a modified linear-quadratic formula. In general, it is very hard to distinguish additive from synergistic model in chemoradiation data (16). *In vitro* experiments can provide some information, but do not necessarily reflect the complex *in vivo* microenvironment. Some chemotherapy drugs used for LAUPC treatment apparently sensitize cell cultures to X-rays (17, 18), but simple additive effects are observed when the drugs are given *in vitro* concomitantly to charged particles (19, 20). Moreover, in many clinical protocols, chemotherapy is given as adjuvant or neo-adjuvant, and even when concomitant is often continued after the radiotherapy cycle. We therefore assumed, in our analysis, that the simple additive model of Eqs 1 and 3.

The BED was calculated using the Fowler formula (11):

(4) $$\text{BED}=\mathrm{nd}\left(1+\frac{d}{\alpha ∕\beta }\right)-\frac{\ln\left(2\right)}{\alpha }\cdot \frac{T}{T_{d}}$$

with:

– *n*: number of fractions
– *d*: dose/fraction
– *T*: overall treatment time
– α = 0.393 Gy^−1^, β = 0.058 Gy^−2^, α/β = 6.77 Gy (21)
– *T*_d_: tumor doubling time, fixed to 42 days (15).

The dose/fraction *d* was given in Gy for X-ray data, and Gy(RBE) (or GyE) for CPT. For protontherapy, 1 Gy(RBE) = 1.1 Gy (22). In CIRT, Gy(RBE) was calculated according to the NIRS model (23), whose results can be different, depending on the dose and target size, from those that would be obtained using the LEM model (24), implemented in the European CIRT facilities.

### Fitting

Clinical data extracted from the published papers were weighted with a vertical error bar, given by the SD of the OS using Poisson statistics:

(5) $$\text{O}{\text{S}}_{1-\text{Year}}=\frac{n_{\text{s}}}{n_{\text{tot}}}\pm \frac{\sqrt{n_{\text{s}}}}{n_{\text{tot}}}$$

where *n*_s_ and *n*_tot_ indicate the number of surviving patients at 1 year and the total number of patients included in the study, respectively. When possible, a horizontal error bar was also included, corresponding to the range of the doses used. A first weighed fit of the radiotherapy-alone data was performed using Eq. 2 to estimate the two parameters γ_50_ and *D*_50_. The chemoradiation data were then fitted using Eq. 3 having CS as only fitting parameter: γ_50_ and *D*_50_ were indeed taken from the radiotherapy fit. Many different chemotherapy drugs were used in old and new studies. Gemcitabine is one of the most successful and currently adopted, also in the CIRT trials. We have therefore divided the data into gemcitabine only, other drugs, and gemcitabine plus other drugs. Overall, no statistically significant differences were noted among the three groups. We have therefore fitted the data together, even if we plotted the points in different colors. Finally, for fitting the CPT data, we expressed the BED in Gy(RBE) as described above, and used Eq. 3 with a fixed CS and γ_50_ taken from the fit of the chemoradiation data with X-rays. In fact, we assume that CPT has an impact on the *D*_50_ due to the putative improved dose distribution in the target and to the radiobiological properties beyond the calculated RBE used in the Gy(RBE).

## Results

### Single treatment data

Chemoradiation is generally considered the best standard of cure for LAUPC. For this reason, only a few studies are available with radiotherapy alone, and some of them are old (Table 1). Some recent studies using SBRT have been excluded. An initial trial in Stanford using high-dose (25 Gy) single-fraction reports a 100% survival at 1 year, but this was limited to six patients (25). Later results from Stanford using SBRT are included in Table 2. On the other hand, a Danish study using 45 Gy in three fractions gave very low OS and high toxicity (26). This study was also excluded in our analysis, because these poor outcomes were likely a result of inaccurate positioning, lack of effective motion management techniques, and lack of dose constraints for OARs (27).

**Table 1:** **Clinical data for treatment of LAUPC using X-ray radiotherapy alone**.

Reference	Year	Total dose (Gy)	Fractions	Sample size	1 year OS	2 years OS	Median OS
Moertel et al. (38)	1969	35–40	20	28	7%	N/A	N/A
Moertel et al. (39)	1981	60	30	25	10%	N/A	5.3 months
Ceha et al. (40)	2000	70–72	35–36	44	39%	N/A	10 months
Cohen et al. (41)	2005	59.4	33	49	20%	N/A	7.1 months
Wang et al. (42)	2015	46	23	14	35%	14%	7.4 months

**Table 2:** **Clinical data for treatment of LAUPC using X-ray therapy plus gemcitabine**.

Reference	Year	Total dose (Gy)	Fractions	Chemotherapy	Sample size	1 year OS	2 years OS	Median OS (months)
Wolff et al. (43)	2001	30	10	Gem, 350–500 mg/m^2^/week for 7 weeks	18	66%	N/A	6
Epelbaum et al. (44)	2002	50.4	28	Gem, 1000 mg/m^2^ weekly before and after RT, Gem 400 mg/m^2^ weekly during RT	20	30%	N/A	N/A
Joensuu et al. (45)	2004	50.4	28	Gem, 20/50/100 mg/m^2^ twice weekly before RT	28	55%	N/A	25
Okusaka et al. (46)	2004	50.4	28	Gem, 250 mg/m^2^ weekly + maintenance 1000 mg/m^2^ weekly for 3 weeks every 4 weeks	38	28%	23%	9.5
Murphy et al. (47)	2007	36	15	Gem, 1000 mg/m^2^ on days 1, 8, and 15	74	46%	13%	11.2
Small et al. (48)	2008	36	15	Gem, 1000 mg/m^2^ 2–3 times/week before, during, and after RT treatment	14	47%	N/A	N/A
Igarashi et al. (49)	2008	40–50.4	20–28	Gem, 40 mg/m^2^ twice/week + maintenance 1000 mg/m^2^ for 3 weeks	15	60%	N/A	15
Schnellenberg et al. (50)	2008	25	1	Gem, 1000 mg/m^2^ weekly for 3 weeks before RT + maintenance weekly	16	50%	N/A	11.4
Polistina et al. (51)	2010	30	3	Gem, 1000 mg/m^2^ weekly for 6 weeks before RT + maintenance weekly	23	39.1%	0%	10.6
Loehrer et al. (52)	2011	50.4	28	Gem, 600 mg/m^2^ weekly before and during RT	34	50%	12%	11.1
Schnellenberg et al. (53)	2011	25	1	Gem, 1000 mg/m^2^ weekly before and after RT	20	50%	20%	11.8
Cardenes et al. (54)	2011	50.4	28	Gem, 600 mg/m^2^ weekly before and during RT + maintenance 1000 mg/m^2^ weekly	28	30%	11%	10.3
Shibuya et al. (55)	2011	54	30	Gem, 250 mg/m^2^ weekly during RT + maintenance 1000 mg/m^2^ every 4 weeks (discretional)	21	74%	N/A	16.6
Mahadevan et al. (56)	2011	24–36	3	Gem, 1000 mg/m^2^ weekly before, during, and after RT (at least 6 cycles)	39	72%	33%	20
Huang et al. (57)	2011	50.4–63	28–35	Gem, 1000 mg/m^2^ weekly during RT + induction/adjuvant (discretional)	55	51%	N/A	12.5
Mukherjee et al. (58)	2013	50.4	28	Gem, induction 300 mg/m^2^ + concurrent 1000 mg/m^2^	38	64.2%	N/A	13.4
Gurka et al. (59)	2013	25	5	Gem, 1000 mg/m^2^ weekly before and after RT	10	50%	N/A	12.2
Herman et al. (60)	2014	33	5	Gem, 1000 mg/m^2^ weekly before and after RT	49	59%	18%	13.9

**Figure 2:**
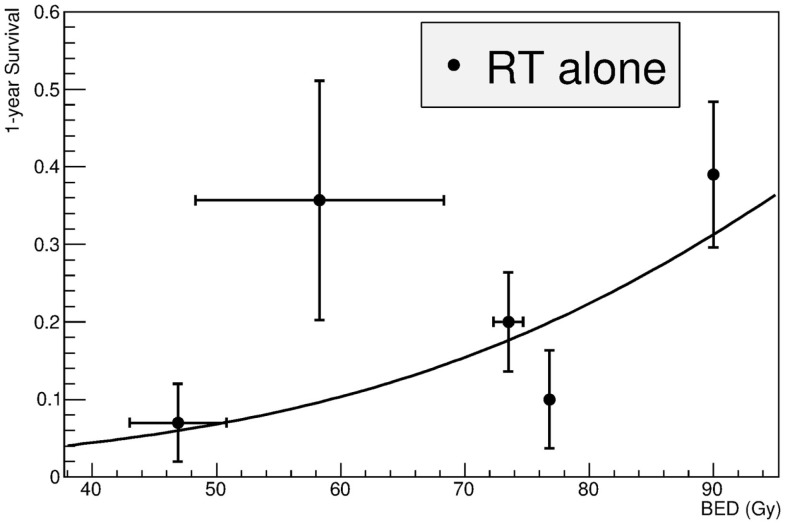
**Fit of the clinical data for treatment of LAUPC with X-ray radiotherapy alone**. Studies are listed in Table 1. BED is calculated by Eq. 4. Fitting was performed by Eq. 2 and fitting parameters are in Table 3.

The data are plotted in Figure 2, along with the fit using Eq. 2. Fitting parameters are reported in Table 2. The *D*_50_ = 107 Gy clearly shows how impractical is the treatment of LAUPC with X-rays alone. For comparison, Dale et al. (16) estimated a BED at 50% complete response for bladder cancer of 54.4 Gy. From the analysis of the trials using chemotherapy alone (10), an average 1-year survival below 20% can be estimated.

### Chemoradiation

**Figure 3:**
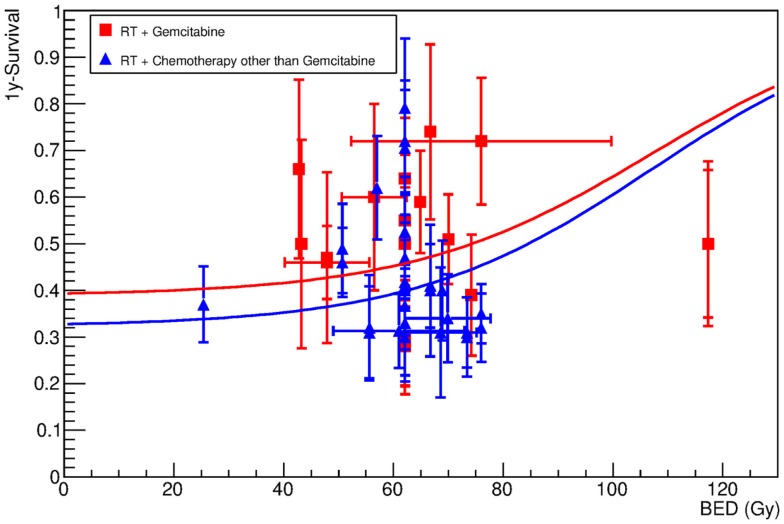
**One-year survival as a function of the BED for patients undergoing X-ray radiotherapy plus gemcitabine (red symbols), or other chemotherapy drugs (blue symbols)**. Data are reported in Tables 2 and 4. The lines show the result of the fit (Eq. 3), which was performed assuming that γ_50_ and *D*_50_ are obtained by fitting the data in treatments using radiotherapy only (Figure 1). The only free fitting parameter is the chemotherapy survival CS (see Table 3). The results suggest that the final outcome does not strongly depend on the specific chemotherapy treatment, although some advantage seems to be associated to the use of gemcitabine.

**Figure 4:**
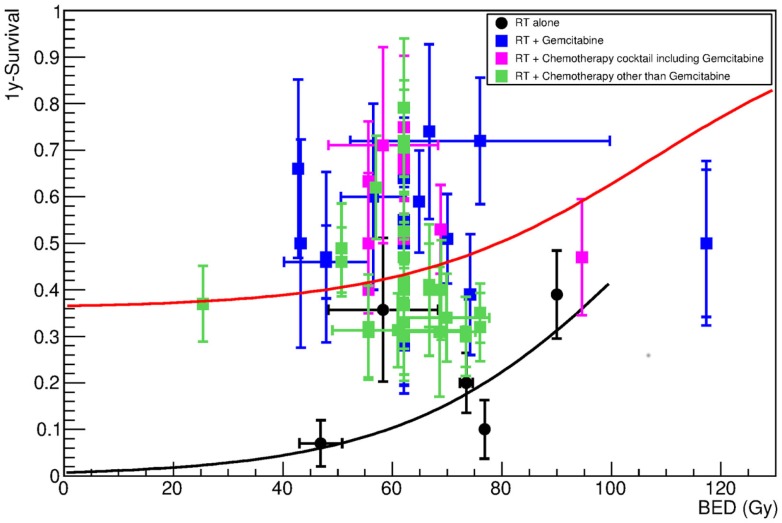
**One-year survival as a function of the BED for patients undergoing X-ray radiotherapy alone (black symbols), or in combination with any chemotherapy treatments**. Details about chemotherapy regimen are reported in Tables 4–6. The lines show the result of the fit (black for radiotherapy-alone data, red for all chemotherapy data pooled together), which was performed assuming that γ_50_ and *D*_50_ are obtained by fitting RT-alone data. Fitting parameters with Eq. 3 are in Table 3.

Meta-analysis of the clinical data has already shown an advantage in chemoradiation compared to radiotherapy or chemotherapy alone (10). Most clinical trials for LAUPC resort to chemoradiation protocols. Gemcitabine (Table 3) is often regarded as the standard treatment. Several other drugs, such as capecitabine, fluorouracil (5-FU), cisplatin, docetaxel, cetuximab, and fluoropyrimidine prodrug S-1, have been used in the past or in new trials (Table 4), and often combination of gemcitabine and any of the other drugs (Table 5) are applied. The standard X-ray course is 50.4 Gy in 1.8 Gy/fraction, giving a BED of 63 Gy. We did not find significant differences in the groups treated with different drugs, considering the very high scatter of the data also due to the completely different protocols adopted. Figure 3 shows, for example, a comparison of the data in Tables 3 and 4, pointing only to a slight trend for better results in protocols using gemcitabine compared to other drugs. Figure 4 shows the fit of all the data compared to X-rays alone. Having fixed the γ_50_ and *D*_50_ parameters, we estimated the only parameter CS = 0.36 ± 0.01 (Table 2). The radiation dose corresponding to this survival probability RS = CS can be estimated by Eq. 2 as

(6) $$\text{BED (chemo – equivalent)=}D_{\text{50}}\left(1-\frac{\ln\frac{1-\text{RS}}{\text{RS}}}{4\gamma_{\text{50}}}\right)$$

leading to a chemo-equivalent dose of 94 Gy. This high value underlines the large improvement that chemotherapy gives on the survival of LAUPC patients. Dale and co-workers (16) estimated 43.6 Gy for the BED chemo-equivalent in bladder cancer. They also demonstrated that the chemo-equivalent dose is not a constant and will be of course much lower if we calculate it for a higher survival level.

**Table 3:** **Fitting parameters calculated using the Eqs 2 or 3**.

Dataset	Table	γ_50_	D_50_ [Gy or Gy(RBE)]	Chemotherapy survival rate (CS)	Figure
Radiotherapy (X-rays) alone	1	1.2 ± 0.5	107 ± 16	N/A	2
Radiotherapy (X-rays) + gemcitabine	2	1.2 (fixed)	107 (fixed)	0.39 ± 0.03	3
Radiotherapy (X-rays) + chemotherapy other than gemcitabine	4	1.2 (fixed)	107 (fixed)	0.32 ± 0.02	3
Radiotherapy (X-rays) + chemotherapy (all protocols combined)	3–5	1.2 (fixed)	107 (fixed)	0.36 ± 0.01	4
CPT + chemotherapy	6	1.2 (fixed)	75 ± 9	0.36 (fixed)	5

**Table 4:** **Clinical data for treatment of LAUPC using X-ray therapy plus chemotherapy, excluding the trials with gemcitabine**.

Reference	Year	Total dose (Gy)	Fractions	Chemotherapy	Sample size	1 year OS	2 years OS	Median OS (months)
Moertel et al. (39)	1981	40	20	5-FU 500 mg/m^2^ 3 days/week during RT, maintenance 5-FU 500 mg/m^2^ weekly	83	46%	N/A	11.4
		60	30		85	35%	N/A	8.4
Wagener et al. (61)	1996	40	20	Epirubicin + Cisplatin + 5-FU	53	49%	N/A	10.8
Ishii et al. (62)	1997	50.4	28	5-FU 500 mg/m^2^ 3 days/week during RT	20	41.8%	N/A	10.3
Fisher et al. (63)	1999	45	25	5-FU 150–250 mg/m^2^ continuous infusion 24 h/day during RT	25	32%	N/A	9
Andre et al. (64)	2000	45	25	5-FU 375 mg/m^2^ + Cisplatin 15 mg/m^2^ daily during RT (first and last week) + maintenance after RT	32	31%	12.5%	9
Boz et al. (65)	2001	59.4	33	5-FU 150–300 mg/m^2^ continuous infusion 24 h/day during RT	42	30%	N/A	9.1
Safran et al. (66)	2001	50.4	28	Paclitaxel 50 mg/m^2^ weekly during RT	44	30%	N/A	8
Li et al. (67)	2003	50.4–61.2	28–34	5-FU 500 mg/m^2^ for 3 days every 2 weeks during RT, Gem 1000 mg/m^2^ after RT	16	31%	0%	6.7
Morganti et al. (68)	2004	39.6–59.4	22–33	5-FU 1000 mg/m^2^ during RT at days 1–4 and 21–24	50	31.3%	N/A	N/A
Cohen et al. (41)	2005	59.4	33	5-FU 1000 mg/m^2^ at days 1–4 and 21–24 + Mitomycin 10 mg/m^2^ at day 2 during RT	55	31%	N/A	8.4
Park et al. (69)	2006	20	10	5-FU 500 mg/m^2^ for 3 days twice during RT with 2 weeks break	56	37%	14.6%	10.4
Chauffert et al. (70)	2008	60	30	5-FU 300 mg/m^2^ 5 days/week for 6 weeks + Cisplatin 20 mg/m^2^ 5 days/week on weeks 1 and 5, maintenance Gem 1000 mg/m^2^ weekly	59	32%	N/A	8.6
Crane et al. (71)	2009	50.4	28	Capecitabine 825 mg/m^2^ twice daily + Bevacizumab 5 mg/kg on days 1, 15, and 29; maintenance Gem 1000 mg/m^2^ weekly + Bevacizumab 5 mg/kg every 2 weeks	82	47%	N/A	11.9
Sudo et al. (72)	2011	50.4	28	S-1 80 mg/m^2^ daily during and after RT	34	70.6%	N/A	16.8
Oberic et al. (73)	2011	54	30	Docetaxel 20 mg/m^2^ weekly + 5-FU 200 mg/m^2^ daily during RT	20	40%	N/A	10
Brunner et al. (74)	2011	55.8	33	5-FU 1000 mg/m^2^ on days 1–5 and 29–33 + Mitomycin 10 mg/m^2^ on days 1–29 during RT	35	40%	N/A	9.7
Huang et al. (57)	2011	50.4–63	28–35	5-FU 200–300 mg/m^2^ 5 days/week or 5-FU 500 mg/m^2^ on days 1–3 and 29–31 or capecitabine 1300–1600 mg/m^2^ daily during RT	38	24%	N/A	10.2
Malik et al. (75)	2012	50.4	28	5-FU based during RT*	84	52.6%	N/A	10.9
Ikeda et al. (76)	2012	50.4	28	S-1 80 mg/m^2^ twice daily during RT, maintenance S-1 80 mg/m^2^ daily after RT	60	72%	N/A	16.2
Schinchi et al. (77)	2012	50	40	S-1 80 mg/m^2^ twice daily during and after RT	50	62%	27%	14.3
Mukherjee et al. (58)	2013	50.4	28	Capecitabine 830 mg/m^2^ 5 days/week induction and concurrent to RT	36	79.2%	N/A	13.4
Herman et al. (78)	2013	50.4	28	5-FU 200 mg/m^2^ daily during RT, maintenance Gem 1000 mg/m^2^ weekly	90	36.7%	10.3%	10
				5-FU 200 mg/m^2^ daily + TNFerade weekly during RT, maintenance Gem 1000 mg/m^2^ weekly	187	41%	11.3%	10
Ducreaux et al. (79)	2014	54	30	Docetaxel 20 mg/m^2^ + Cisplatin 20 mg/m^2^ weekly during RT	51	41%	31%	9.6
Rembielak et al. (80)	2014	50.4	28	Cetuximab loading dose 400 mg/m^2^ + 250 mg/m^2^ weekly during RT	21	33%	11%	7.5
Kwak et al. (81)	2014	50.4	28	5-FU 600–1000 mg/m^2^ during RT, maintenance Gem 200 mg/m^2^ weekly	34	40%	10%	9

*Limited information about chemotherapy.

**Table 5:** **Clinical data for treatment of LAUPC using X-ray therapy plus a chemotherapy cocktail including gemcitabine**.

Reference	Year	Total dose (Gy)	Fractions	Chemotherapy	Sample size	1 year OS	2 years OS	Median OS
Chung et al. (82)	2004	45	25	Gem 1000 mg/m^2^ weekly + Doxifluoridine 600 mg/m^2^ daily during and after RT	22	50%	N/A	12
Haddock et al. (83)	2007	45	25	Gem 30 mg/m^2^ + Cisplatin 10 mg/m^2^ twice weekly during first 3 weeks of RT, Gem 1000 mg/m^2^ weekly after RT	48	40%	N/A	10.2
Hong et al. (84)	2008	45	25	Gem 1000 mg/m^2^ weekly + Cisplatin 70 mg/m^2^ two times during RT, maintenance Gem 1000 mg/m^2^ weekly + Cisplatin 70 mg/m^2^ every 4 weeks	38	63.3%	27.9%	16.7
Mamon et al. (85)	2011	50.4	28	Gem 200 mg/m^2^ weekly + 5-FU 200 mg/m^2^ 5 days/week during RT, maintenance Gem 1000 mg/m^2^ weekly	78	51%	N/A	12.2
Crane et al. (86)	2011	50.4	28	Gem 1000 mg/m^2^ + Oxaliplatin 100 mg/m^2^ before RT + Capecitabine 825 mg/m^2^ twice daily on RT days, cetuximab 500 mg/m^2^ every 2 weeks before and during RT	69	66%	25%	19.2
Brunner et al. (74)	2011	55.8	31	Gem 300 mg/m^2^ + Cisplatin 30 mg/m^2^ weekly during RT	58	53%	N/A	12.7
Ch’Ang et al. (87)	2011	50.4	28	Gem 800 mg/m^2^ + Oxaliplatin 85 mg/m^2^ + 5-FU/Leucovorin 3000/150 mg/m^2^ twice/week before RT, Gem 400 mg/m^2^ weekly during RT	50	68%	20.6%	14.5
Tozzi et al. (88)	2013	45	6	Gem-based before RT	30	47%	N/A	11
Ke et al. (89)	2014	50.4	28	Gem 1000 mg/m^2^ weekly + S-1 40 mg/m^2^ twice daily before RT, S-1 80 mg/m^2^ twice daily during RT, S-1 80 mg/m^2^ twice daily 1 month after RT	32	75%	34.4%	15.2
Wang et al. (42)	2015	46	23	Gem-based (sub-groups)	16	71.1%	40.6%	19.5

### Charged particle therapy

**Figure 5:**
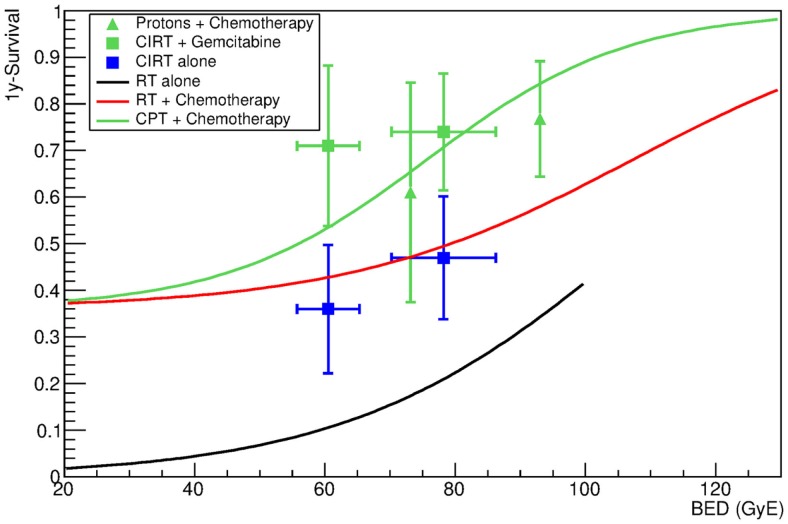
**One-year survival as a function of the BED for patients undergoing CPT with or without additional chemotherapy**. Blue symbols refer to patients receiving radiotherapy with C-ions without additional chemotherapy. Green symbols refer to data obtained with proton (triangles) and carbon ions (full squares) in combination with chemotherapy. Data are given in Table 6. The green line shows the result of the fit of data for chemotherapy combined with proton or carbon ions. The fit was performed using γ_50_ and CS from X-ray + chemotherapy data. The only free parameter is therefore *D*_50_. The black and red lines show the results of the fit for X-rays alone and X-rays plus chemotherapy, and are reported for comparison. Fitting parameters are in Table 3.

Although only a few studies are available with CPT, the data in Table 6 show that they are the best current options for LAUPC. A 2-year survival rate around 50% was reached with protons (28) or C-ions (9) in combination with gemcitabine, a value far exceeding any other chemoradiation trial using X-rays and any cocktail of drugs. The data with CIRT alone (no chemotherapy) are clearly superior to those with X-rays alone and comparable to the results with chemoradiation at the same X-rays BED. The best 1-year OSs for combined chemotherapy (gemcitabine) and CPT are those from Hyogo (28) using protons up to 70.2 Gy(RBE) in 26 fractions, but they came at a cost of grade 3–5 toxicity in 10% of the patients, especially gastric ulcer and hemorrhage. CIRT toxicity was much more mild, with 17% of the patients experiencing grade 3 GI toxicity, in the form of appetite loss. Low toxicity was observed for the duodenum, both for protons and ^12^C-ions. The fit of the chemoradiation with CPT, using the same CS and γ_50_ parameters calculated for X-rays + chemotherapy, is shown in Figure 5. This fit assumes that CPT does not change the effect of the chemotherapy compared to X-rays, but results in a lower D_50_ due to biological and/or physical improvements compared to X-rays. Should these improvements be already included in the RBE model used to calculate the equivalent dose in Gy(RBE), we should see the same effect at the same BED [see Ref. (23) for CIRT in Japan; RBE = 1.1 for protons]. Instead, the best fit is reduced to *D*_50_ = 75 ± 9 Gy(RBE) for CPT (Table 2). This 50% improvement is caused either by a better physics, enabling treatment of infiltrations in the neuroplexus, or to a better biology, especially to a reduced OER (6) or to a stronger immune response (7) using CPT compared to X-rays.

**Table 6:** **Clinical data for treatment of LAUPC using CPT**.

Reference	Year	Radiation quality	Total dose in Gy (RBE)	Fractions	Chemotherapy	Sample size	1 year OS	2 years OS	Median OS
Terashima et al. (28)	2012	Protons	67.5	25	Gem, 800 mg/m^2^/week for 3 weeks	50	76.8%	50%	N/A
Sachsman et al. (90)	2014	Protons	59.4	33	Capecitabine, 1000 mg twice/day; 5 days/week on radiation treatment days only	11	61%	31%	18.4
Yamada et al. (9)	2014	Carbon ions	38.4–43.2	12	–	19	36%	5%	N/A
					Gem 1000 mg/m^2^/week for 3 weeks	24	71%	21%	N/A
			45.6–52.8	12	–	27	47%	16%	N/A
					Gem 1000 mg/m^2^/week for 3 weeks	47	74%	54%	N/A

## Discussion

**Figure 6:**
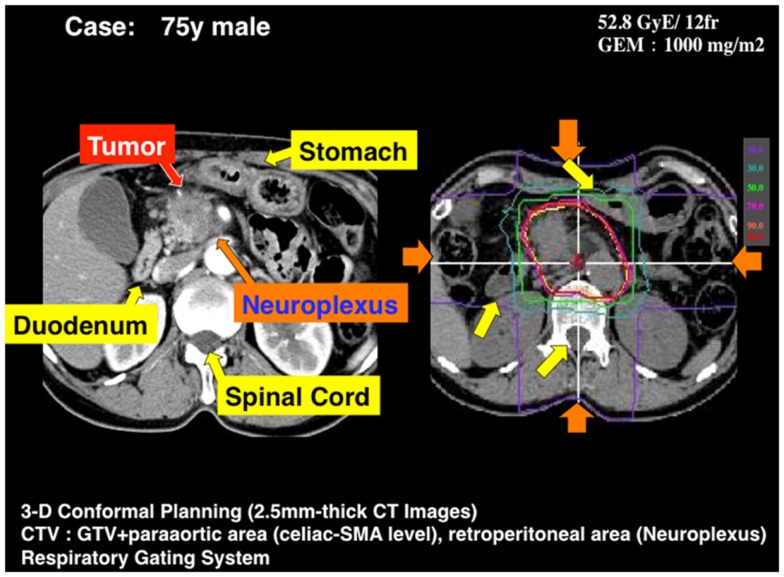
**A typical treatment plan used at NIRS for a locally advanced pancreatic head cancer**. The beam is shaped with passive modulation and four opposite fields are applied with respiratory gating. GTV includes the primary tumor and lymph nodes involved. CTV = PTV + neuroplexus infiltration (periarterial area) + proximal lymph nodes. PTV = CTV + 5 mm, excluding GI tract.

**Figure 7:**
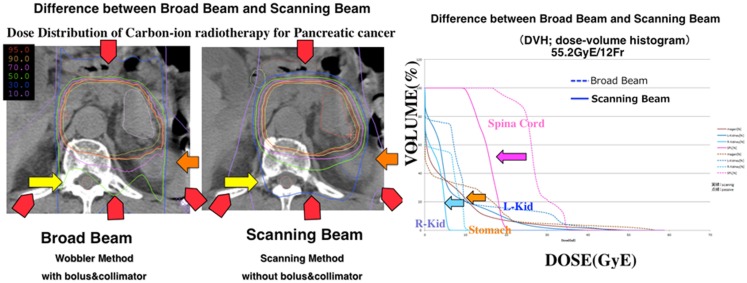
**Comparison of the current passive beam modulation treatment plan with a spot scanning treatment plan for LAUPC**. In the right panel, the dose–volume histogram for different organs is shown for passive modulation (dotted line) and raster scanning (solid line). Dose to the spinal cord and kidney are highly reduced. Potential reduction is also clear for stomach and duodenum, whose movements are, however, critical.

The large interest for the use of CPT in LAUPC comes from the exceptional clinical results (8), supported by our clinical data analysis in Figure 5. These results reflect the biological rationale of reduced OER for high-LET radiation and possible dose escalation with limited side effects exploiting the Bragg peak. The high GI toxicity observed in the Hyogo trial (28) seems to set a threshold at a BED around 100 Gy(RBE). The question is whether the same threshold applies to CIRT, where the sharper dose edges of the treatment plan may reduce the exposure of the critical organs compared to protons, whose lateral scattering is much higher than for heavy ions (6). An example of a treatment plan of a pancreatic head cancer with carbon ions is shown in Figure 6. It is possible to give a high-dose against tumor and neuroplexus with acceptable doses to stomach or duodenum. The dose distribution can further improve using raster scanning instead of passive modulation, as shown in Figure 7. The new NIRS facility is now equipped with raster scanning, and so are the HIT and CNAO facilities now treating the first LAUPC patients with C-ions. Under these optimal conditions, it appears feasible to exceed a BED of 100 Gy(RBE) with acceptable toxicities.

**Figure 8:**
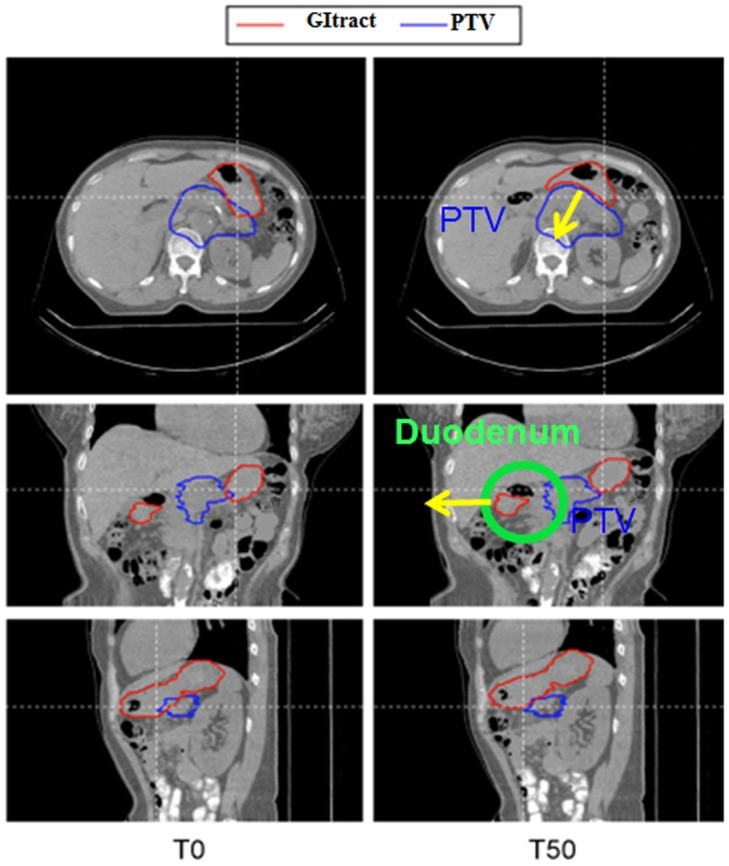
**4DCT analysis of the movement of the critical organs during treatment of LAUPC at NIRS with C-ions**. T0 is the peak inhalation and T50 the peak exhalation phases. Stomach and duodenum move in and out the PTV in the two phases.

Modeling chemotherapy in terms of equivalent radiation dose is an effective method to predict outcomes of dose-escalation trials (12, 16). The large scatter in the chemoradiation data leads, however, to a poor goodness-of-fit in Figures 3 and 4. This is due in part to the many different protocols used in chemotherapy for LAUPC, and to inclusion of data published in over 30 years using very different methods both for drug and radiation delivery. In this paper, we have decided to analyze all the data available in the literature, without including the treatment year as a function in the model. We have also assumed no synergistic interaction between chemicals and radiation. Finally, Eq. 4 should be modified for protons or carbon ions, where α/β is higher than for X-rays leading to a lower dependence on fractionation. Due to the lack of sufficient information leading to an educated guess for other parameters and models, we decided to stick to the conventional logistic function, replacing Gy with Gy(RBE) in Table 6. The basic assumption remains that a higher BED will result in a higher OS in LAUPC patients, an assumption clearly supported by the analysis of the several trials included in our data mining. Our analysis supports the concept that a dose escalation will improve OS, and toxicity is the limiting factor. In Table 7, we have calculated with the logistic model (Eq. 3) the expected survival in hypofractionated dose-escalation trials and compared with the standard chemoradiation treatment and other schedules proposed for SBRT using X-rays (15, 27). The standard at NIRS is 12 fractions in 3 weeks, and with the current maximum dose/fraction the OS at 1 year is expected to improve from 40 to 70% compared to the standard X-ray regime (50.4 Gy in 28 fractions). Reaching 18 fractions with the same dose/fraction, it could be possible to double the survival. Further hypofractionation, down to a single dose of 25 Gy(RBE) is very attractive in terms of expected survival, but raises concerns for the GI toxicity. C-ions delivered by raster scanning should provide the optimal dose distributions (Figure 7) compared to CIRT with passive scattering and protons, where the lateral scattering unavoidably leads to a dose penumbra around the PTV. However, for beam scanning, the issue of motion mitigation must be tackled very carefully, because of the known problem of the interplay. Currently, NIRS is using respiratory gating to compensate especially the movements of stomach and duodenum in the PTV (Figure 8). A treatment with high number of fractions compensates the interplay between beam scanning and organ motion, but this compensation is lost in radiosurgery (29). In the treatment of hepatocellular carcinoma with ^12^C-ions at the HIT facility in Heidelberg, it has been shown that the simple increase from 1 to 4 fractions substantially improved the dose target coverage and reduced overdosage (V107 from 32 to 4%) (30), this means that keeping the hypofractionation schemes above 4 fractions, major inhomogeneities should be avoided. Nevertheless, the range uncertainties due to bowel movement, stomach peristalsis, and breathing, have to be solved to reduce toxicity to the many critical organs surrounding the pancreas. Motion mitigation strategy include respiratory gating or layer stacking boost irradiation, such as used at NIRS for treating PC (31), and 4D optimization of the plan based on 4DCT (32). Patients with tumors in a favorable location, preferably >1 cm from the closest luminal organ, should be selected for the dose escalation.

**Table 7:** **Expected improvement in survival according to our model in chemoradiation trials using CPT**.

Dose/fraction inGy or Gy(RBE)	Radiation quality	Fractions	Total dose in Gy or Gy(RBE)	BED in Gy or Gy(RBE)	Expected 1 year survival rate	Comments
1.8	X-rays	28	50.4	62.9	42%	Current standard fractionation scheme
2.25	X-rays	33	74.3	97.8	61%	Proposed dose-escalation trial at Medical College of Winsconsin (15)
6.6	X-rays	5	33	65.2	45%	Standard for SBRT in adjuvant settings (27)
2.7	Protons	26	70.2	97.4	75%	Maximum dose reached at Hyogo
4.6	C-ions	12	55.2	92.5	71%	Maximum dose reached at NIRS
5.85	C-ions or protons	12	70.2	130.6	82%	Maximum total dose reached with protons in Hyogo using the number of fraction from NIRS
25	C-ions or protons	1	25	117.5	76%	Maximum dose used in single-fraction X-ray radiosurgery for LAUPC (27)
4.6	C-ions or protons	18	82.8	138.6	84%	Expected doubling of the OS with conventional X-ray fractionation scheme, using the dose/fraction from NIRS

*BED is calculated by Eq. 4. Expected 1 year survival is calculated using Eq. 3 and the parameters in Table 3*.

The solution of this problem is an important step to push toward higher doses and fewer fractions thus leading to a substantial improvement in survival can be expected using chemoradiation protocols with CPT rather than X-rays. The first clinical CIRT vs. IMRT trial for LAUPC should compare the standard chemoradiation treatment (Table 7, row 1), with the NIRS most advanced protocol (Table 7, row 5). The additional advantage of using the standard protocols is that at the dose/fraction of 4.6 Gy(RBE) reached in the escalation trial at NIRS, there is practically no difference between the biological dose calculated at NIRS and those predicted by LEM (24) and implemented in European CIRT facilities. However, in a multi-centric trial, it will be unavoidable to have different systems for dose delivery, motion management, patient selection, etc. For instance, NIRS is using passive modulation, CNAO raster scanning, and HIT can use the gantry. Nevertheless, a comparative trial for LAUPC is absolutely necessary to support the use of CIRT and to confirm the very promising data in the phase I–II trials at NIRS (8). The lack of comparative, phase-III clinical trials is generally considered as a major hindrance to a more widespread use of CPT in the clinics (33). A trial on LAUPC may definitely clarify the clinical advantage of CPT in such a lethal tumor.

Apart from the international comparative trial, further developments of phase-II trials with CPT should point to two directions. First, several molecular markers, such as mutations in *SMAD4/DPC4*, have been validated as prognostic factors in PCs (34). Whole-genome sequencing and copy number variation analysis suggest that PCs can be divided into four genetic subtypes, with potential clinical utility (35). Trials with CPT combined with molecular analysis of these genes are highly needed, because CPT may elicit different molecular pathways than conventional X-rays (36). Combined CIRT + gemcitabine may be especially effective against pancreatic stem-like cells, as suggested by a recent *in vitro* study (37), and hence, study of stem cells markers and genetic pathways will be highly desirable. In addition, further hypofractionation is desirable if the problems of the organ movements are tackled as described above. For instance, the use of 12 fractions (such as done at NIRS) with the total dose used for protons in Hyogo is expected to push the 1-year survival over 80% (Table 7, row 6). A careful motion mitigation strategy should be rapidly implemented to allow this further escalation.

## Conflict of Interest Statement

The authors declare that the research was conducted in the absence of any commercial or financial relationships that could be construed as a potential conflict of interest.

## Footnotes

^1^Solicitation number BAA-N01CM51007-51 (April 17, 2015) available online through FedBizOpps at http://www.fbo.gov
